# Sex differences in neural stress responses and correlation with subjective stress and stress regulation

**DOI:** 10.1016/j.ynstr.2019.100177

**Published:** 2019-05-25

**Authors:** Elizabeth V. Goldfarb, Dongju Seo, Rajita Sinha

**Affiliations:** a Department of Diagnostic Radiology, Yale School of Medicine, New Haven, CT, USA; b Yale Stress Center, Yale School of Medicine, New Haven, CT, USA; c Department of Psychiatry, Yale School of Medicine, New Haven, CT, USA; d Department of Neuroscience, Yale School of Medicine, New Haven, CT, USA

**Keywords:** Stress, Sex, Emotion, fMRI, Medial prefrontal cortex, Hippocampus

## Abstract

Emotional stress responses, encompassing both stress reactivity and regulation, have been shown to differ between men and women, but the neural networks supporting these processes remain unclear. The current study used functional neuroimaging (fMRI) to investigate sex differences in neural responses during stress and the sex-specific relationships between these responses and emotional stress responses for men and women. A significant sex by condition interaction revealed that men showed greater stress responses in prefrontal cortex (PFC) regions, whereas women had stronger responses in limbic/striatal regions. Although men and women did not significantly differ in emotional stress reactivity or subjective reports of stress regulation, these responses were associated with distinct neural networks. Higher dorsomedial PFC responses were associated with lower stress reactivity in men, but higher stress reactivity in women. In contrast, while higher ventromedial PFC stress responses were associated with worse stress regulation in men (but better regulation in women), dynamic increases in vmPFC responses during stress were associated with lower stress reactivity in men. Finally, stress-induced hippocampal responses were more adaptive for women: for men, high and dynamically increasing responses in left hippocampus were associated with high stress reactivity, and dynamic increases in the left (but not right) hippocampus were associated with worse stress regulation. Together, these results reveal that men and women engage distinct neural networks during stress, and sex-specific neural stress responses facilitate optimal emotional stress responses.

## 1 Introduction

Negative and uncontrollable events, or stressors, trigger multiple affective and cognitive responses. These include subjective feelings, or *stress reactivity*, which help signal that the organism is in a stressful situation, as well as *stress regulation*, which supports cognitive, emotional and behavioral coping to address the distress, the stressor itself and learning to build resilience and adaptation (Sinha, 2008). Thus, both emotional reactivity and timely, flexible modulation of these reactions are adaptive (Gratz and Roemer, 2004; Hartley and Phelps, 2010) and may facilitate optimal responding to stressors to build resilience. Research on stress and emotion processing has highlighted the neural circuitry supporting these responses. For example, early reactivity to acute stressors has been associated with increased signal (measured using functional neuroimaging) in the “salience network”, encompassing subcortical and limbic regions including the amygdala, anterior insula, and striatum (Hermans et al., 2014; van Oort et al., 2017). Connectivity within this network during stress was positively associated with negative affect (Hermans et al., 2011) and these regions are also involved in the generation of emotional responses (Ochsner et al., 2012). The hippocampus has also been associated with emotional reactivity (Kober et al., 2008; Phelps, 2004), stress-related health issues (Seo et al., 2014) and regulation, particularly of the physiological components of the stress response (Herman et al., 2012). In contrast, the medial prefrontal cortex (mPFC; especially ventromedial prefrontal cortex, vmPFC), which has strong inhibitory projections to the amygdala (Quirk et al., 2003; Wood et al., 2019), has been associated with a variety of processes that may promote stress coping. These include recognizing that a stressor can be controlled (Maier, 2015); knowing that a previously threatening situation is now safe (“fear extinction”; Milad and Quirk, 2012); resilient coping (Maier and Watkins, 2010) and, more broadly, integrating the current context and goals with emotional valuation (Ochsner et al., 2012). A recent study directly linked dynamic increases in vmPFC responses during stress to higher levels of self-reported active coping strategies in humans (Sinha et al., 2016). Together, this corticolimbic network has been proposed to underlie sex differences in the negative consequences of stress exposure (Bangasser and Valentino, 2014).

Although stress reactivity and regulation are core aspects of the stress response, sex differences in these processes have been reported. These differences have significant public health implications, as men and women also differ in their risks for stress-related psychopathology (Bangasser and Valentino, 2014; Becker et al., 2007; Brady and Sinha, 2005). With regard to stress reactivity, women often self-report higher levels of subjective distress in response to an acute stressor (Childs et al., 2010; Kelly et al., 2008; Steptoe et al., 1996). In contrast, men tend to have stronger physiological stress reactivity, as indexed by increases in levels of glucocorticoid hormones (Childs et al., 2010; Kinner et al., 2014; Kirschbaum et al., 1992; Steptoe et al., 1996), although the opposite pattern has been reported in rodents (Bale and Epperson, 2015). Theoretical and empirical work suggest that men and women engage qualitatively different stress coping strategies (Matud, 2004; Nolen-Hoeksema, 2012; Tamres et al., 2002; Taylor et al., 2000). These differences may reflect distinct evolutionary priorities (Shansky, 2018). Females have been proposed to preferentially engage a “tend-and-befriend” response to stressors, whereas males are more likely to express a “fight or flight” response (Taylor et al., 2000). This is consistent with research in rodents showing that females often engage passive coping strategies whereas males prefer active coping (Bale and Epperson, 2015), although this pattern can vary by stressor (Hodes, 2018) and available responses (Gruene et al., 2015). In humans, women have reported greater use of most coping strategies, particularly those that involve verbal expression of emotions (Tamres et al., 2002).

This sex-specific variability in emotional stress responses may be associated with differences in neural responses to acute stressors between men and women. Several recent studies have shown that men and women vary in their stress responses within the same corticolimbic/striatal circuitry associated with stress reactivity and coping. For example, men had higher vmPFC responses during stress than women (Goldstein et al., 2010; Seo et al., 2011), whereas women showed higher responses in the amygdala (Kogler et al., 2015a), insula, and putamen (Wang et al., 2007), although results from these limbic/striatal regions have been mixed (Kogler et al., 2015a; Seo et al., 2011). In addition to differences in the networks engaged during stress, it is also possible that these regions play different roles in emotional stress responses for men and women. For example, greater damage to mPFC was associated with worse self-concept of abilities during a psychological stressor in men, but not women (Buchanan et al., 2010). In addition, responses in dorsomedial PFC (dmPFC) were positively correlated with stress-induced anxiety for women, but negatively correlated with stress-induced anxiety for men (Seo et al., 2017). Thus, there is a need for research explicitly testing how sex-specific neural responses during stress relate to stress reactivity and regulation for men and women.

In this study, we used functional magnetic resonance imaging (fMRI) procedures to investigate sex-specific responses throughout the brain during an acute, sustained stressor. This protocol, in which a barrage of novel, unpredictable, uncontrollable and highly aversive stimuli are presented per minute across several minutes, has been shown to evoke robust stress responses (Sinha et al., 2016). Here stress reactivity was measured as self-reported stress levels during the fMRI scan, and stress regulation was assessed prior to the scan using a validated questionnaire that measures difficulties across multiple dimensions of emotion regulation (Gratz and Roemer, 2004). We hypothesized that the corticolimbic/striatal regions described above would show distinct responses during the stressor for men and women. We further hypothesized that responses in these regions would be associated with stress reactivity and regulation, but that these relationships would differ between men and women.

## 2 Methods

### 2.1 Participants

Sixty right-handed healthy volunteers completed the study (31 female and 29 male, demographic characteristics shown in Table 1). Participants were screened to ensure they did not meet any of the following exclusion criteria: meeting current criteria for dependence on another psychoactive substance, excluding nicotine; regular use of anticonvulsants, sedatives/hypnotics, prescription analgesics, other antihypertensives, anti-arrythmics, antiretroviral medications, tricyclic antidepressants, SSRIs, naltrexone, or antabuse; current use of opiates or past history of opiate abuse/dependence; psychotic or otherwise severely psychiatrically disabled (i.e., suicidal, homicidal, current mania); significant underlying medical conditions (such as a history of seizure disorder, cerebral, renal, thyroid or cardiac pathology); claustrophobia or ferromagnetic metal in the body (for MRI safety); and, for female participants, pregnant or nursing. All participants were light, non-binging drinkers as defined by National Institute on Alcohol Abuse and Alcoholism (NIAAA) criteria, and only two participants (1 male, 1 female) smoked cigarettes. Of the female participants, 18/31 (58.1%) were not taking any form of contraceptive. As oral contraceptives may influence stress responses (Mordecai et al., 2017), supplemental analyses compared female participants taking no contraceptives to those taking oral contraceptives (8/31). These preliminary analyses indicated that patterns of neural and emotional stress responses, as well as associations between neural and emotional stress responses, were largely consistent across these groups (Fig. S1). Male and female participants did not differ significantly in age, IQ, drinking behavior or levels of perceived stress in the past month (PSS; (Cohen et al., 1983). Participants reported mild levels of anxiety (Hamilton Anxiety Rating Scale scores ≤ 18), although females reported higher anxiety than males (F: mean = 6.1 [SD = 4.91], M: 3.59 [3.99], *t*(57) = 2.16, *p* = .035).

**Table 1:** Demographics.

	All (N = 60)	Female (N = 31)	Male (N = 29)	Difference?
Age	29.1 (9.42)	29.68 (10.05)	28.48 (8.84)	*p* > .25
IQ: Shipley	113.81 (7.01)	113.17 (6.91)	114.48 (7.17)	*p* > .25
AUDIT: Total	3.20 (2.06)	2.81 (1.78)	3.64 (2.28)	*p* = .13
PSS	17.98 (8.81)	18.13 (8.14)	17.83 (9.61)	*p* > .25

Values shown are Mean (SD). AUDIT = Alcohol Use Disorders Identification Test (Babor, de la Fuente, Saunders and Grant, 1992); PSS = Perceived Stress Scale.

### 2.2 Assessments of emotional stress responses

*Stress reactivity.* Emotional responses were measured in response to a sustained laboratory stressor exposure (3.2). Participants rated how stressed they felt when viewing the pictures on a scale from 1 (Not at All) to 9 (Extremely stressed). Participants had 3 s to make these ratings, which were completed during the fMRI scan using a magnet-safe button box and repeated after every 1 min of image exposure.

*Stress regulation*. Participant's perceptions of ability to cope with stress was measured using the Difficulties in Emotion Regulation Scale (DERS) (Gratz and Roemer, 2004). This well-validated 36-item instrument is designed to assess self-reports of difficulties coping with, or regulating, emotions with participants indicating how much each item applies to them on a scale from 1 (Almost Never) to 5 (Almost Always). These are summed across items to yield a “total” emotion regulation score, with higher values indicating greater difficulties. The DERS also provides scores on six subscales: 1) nonacceptance of emotional response (nonacceptance); 2) difficulties engaging in goal-directed behavior (goals); 3) impulse control difficulties (impulse); 4) lack of emotional awareness (awareness); 5) limited access to emotion regulation strategies (strategies); and 6) lack of emotional clarity (clarity). This metric thus enables the assessment of the extent to which individuals are aware of (e.g. (Ma et al., 2017), and accept their emotional reactivity, as well as their perceived ability to control these feelings. Scores on the DERS, together with stressor exposure, have been shown to predict serious stress-related health consequences such as cardiovascular disease (Roy et al., 2018). The questionnaire had high internal consistency in the current sample (Cronbach's α = 0.94).

### 2.3 Stressor exposure

Participants were exposed to a sustained stressor using a block design as described previously (Sinha et al., 2016). Briefly, participants passively viewed a series of visual images in Neutral and Stress conditions (order counterbalanced). Each condition consisted of 8 contiguous runs, each lasting 66 s: 2 baseline runs (5 s gray screen with fixation cross with 1 s inter-stimulus interval [ISI]) followed by 6 visual image runs (each image displayed for 5 s with 1 s ISI). Images in the Neutral condition were based on commonly experienced neural/relaxing situations, such as nature scenery (forest, beach, grass), laying and relaxing on the beach or reading at the park. Images in the Stress condition were highly aversive images (e.g., violence, terror, fear) selected from the International Affective Picture System (Lang et al., 2008) with an average valence rating of 2.34 (SD = 0.63; scale: 1 = negative, 9 = positive) and arousal rating of 6.0 (SD = 0.83; 1 = calm/relaxed, 9 = excited). Emotional intensity and content (i.e., category of images) was equivalent in each run within condition with no statistical difference in valence and arousal normative ratings. Within each run, there were equivalent proportions of social vs nonsocial images and, for social images, equivalent proportions of males and females. Stimuli were presented using EPrime software and were projected onto a screen that participants viewed using a mirror attached to the head coil. To control for circadian fluctuations in physiological stress responses, all scans were run at approximately the same time of day (8:00–10:00 a.m.).

### 2.4 Functional neuroimaging (fMRI) data acquisition

Images were obtained using 3T Siemens MRI systems (Trio and Prisma). Acquisition parameters were the same across scanners, and there was no significant difference in the proportion of male and female participants who were collected using each scanner (female, 77.4% Prisma; male, 82.7% Prisma, χ^2^[df = 1] = 0.038, *p* > .25). Structural data were acquired first using a sagittal high-resolution T1-weighted 3D MPRAGE sequence (TR = 2400 ms, TE = 1.96 ms, flip angle = 8°, FOV = 256 mm × 256  mm, matrix = 256 × 256, 208 slices, 1 mm^3^ isotropic voxels). Functional data were acquired using a multiband gradient echo-planar image sequence (TR = 1000 ms, TE = 30 ms, flip angle = 55°, FOV = 220 mm × 220  mm, 75 slices, interleaved acquisition, 2 mm^3^ isotropic voxels). The multiband technique shortens acquisition time without decreasing TE or sacrificing SNR by simultaneously exciting 5 slices per multiband RF pulse. The scanner waited 4 s at the start of each run prior to acquiring data to allow for scanner stabilization.

### 2.5 Procedure

Participants were instructed not to consume alcohol or other drugs for 48 h prior to the fMRI scan. Starting at 10:00 p.m. the night before the scan, they were told not to eat or drink anything except water and were encouraged to get a good night's sleep. On the day of the fMRI scan, all participants arrived at the Yale Stress Center at 7:15 a.m. and were trained on the fMRI experimental procedure. Outside the scanner, participants practiced rating their subjective stress levels, viewing 16 neutral images that were not repeated during fMRI scan. All participants blew a negative breathalyzer (blood alcohol content < 0.08) confirming no recent alcohol use prior to the start of the scan. Repeated ratings of subjective stress were obtained throughout the scan. In a separate session at the Yale Stress Center, participants completed the DERS questionnaire. Participants provided written informed consent to complete the study and all procedures were approved by the Yale Medical School Institutional Review Board.

### 2.6 Analysis

#### 2.6.1 Emotional stress responses

To test whether males and females differed in stress reactivity, we used a repeated measures analysis of variance (rmANOVA) to analyze ratings of subjective stress throughout the scan. The rmANOVA included Condition (stress vs. neutral) and Time (early/mid/late, relative to their respective baseline) as within-subjects factors and Sex (women vs. men) as a between-subjects factor. We compared stress regulation (DERS scores) between males and females using independent-samples t-tests. One participant (female) did not complete the DERS and was excluded from all analyses with this measure.

#### 2.6.2 fMRI preprocessing

All fMRI scans underwent the same preprocessing steps using FSL and AFNI. Data were high-pass filtered at 0.01 Hz to remove low-frequency drifts in signal, and linear (6 degrees of freedom) and nonlinear motion (FSL's Motion Outliers algorithm) was estimated. Any runs with motion (estimated using MCFLIRT) greater than an absolute mean displacement of 1.5 mm were discarded (2 runs total). A general linear model was then conducted for each run to additionally control for motion-related confounds and covariates of no interest (using FEAT; Woolrich et al., 2001). These regressors included the 6 linear estimated motion parameters, white matter and cerebrospinal fluid timeseries (each plus their temporal derivatives), as well as stick function regressors for nonlinear motion outliers. The residuals from this model were then aligned to a reference functional scan, and then to the participant's high-resolution anatomical scan using boundary based registration (Greve and Fischl, 2009). The images were then warped to MNI space and smoothed to 6 mm FWHM (using 3dBlurToFWHM, which iteratively blurs a timeseries using a diffusion-based approach and estimates a mixed-model autocorrelation function). This approach has been shown to help address motion confounds (Scheinost et al., 2014). These smoothed data were then concatenated into Baseline (Gray runs 1 and 2), Early (Condition runs 1 and 2), Mid (Condition runs 3 and 4), and Late (Condition runs 5 and 6) epochs (following Sinha et al., 2016), and the average BOLD response for each epoch was computed. Finally, the difference relative to Baseline for each Condition epoch (Early-Baseline, Mid-Baseline, Late-Baseline) was computed for each participant and condition (Stress and Neutral).

#### 2.6.3 fMRI analysis

Second level analyses of the preprocessed fMRI data used a linear mixed effects model (3dLME) with Time (early/mid/late), Condition (stress vs. neutral) and Sex (female vs. male) as predictors and Participant as a random effect. To control for multiple comparisons, data were cluster-corrected using the latest version of 3dClustSim, which fits a mixed-model spatial autocorrelation function to model the noise in the fMRI data (Cox et al., 2017). For voxelwise *p* < .01, we used bi-sided first-nearest neighbor clustering to determine a cluster threshold size for α = 0.05. Regions were identified using the Harvard-Oxford Cortical and Subcortical Atlases from FSL, and Brodmann Areas were defined using the Yale BioImage Suite Package web application (Lacadie et al., 2008).

#### 2.6.4 Functional regions of interest (ROIs)

From the whole-brain analysis of regions showing a significant Condition × Sex interaction, we identified clusters from the *a priori* corticolimbic/striatal network associated with stress reactivity and regulation (described in the Introduction) as ROIs. These clusters included regions of medial prefrontal cortex (R dmPFC, L subgenual anterior cingulate cortex [sgACC], L BA 11), R insula/putamen, L pallidum, and bilateral hippocampus. We extracted the mean BOLD signal (averaged across all voxels within each ROI) per participant during Stress relative to Baseline runs.

#### 2.6.5 Relating brain stress response to emotional stress responses

We ran linear models in which Sex (female vs. male) and BOLD response (average response to Stress-Gray for each ROI) were used to predict emotional stress responses. These were run separately for stress reactivity (average ratings of subjective stress during the scan) and stress regulation (total score on DERS). If there was a significant interaction predicting total DERS scores, each DERS subscale was then examined separately. As an exploratory analysis, we also assessed whether dynamic changes in BOLD responses during the stress condition (Late – Early, relative to baseline), previously associated with resilient coping (Sinha et al., 2016), were associated with dynamic changes in stress reactivity (also Late – Early) or overall stress regulation abilities.

## 3 Results

### 3.1 Emotional stress responses: stress reactivity and regulation

**Fig. 1:**
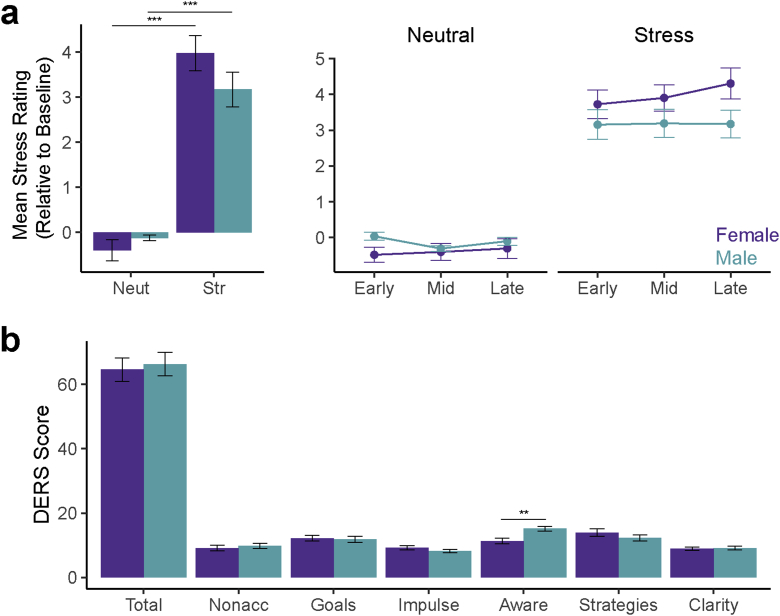
Emotional stress responses from male and female participants. (a) Stress reactivity, as measured by subjective stress ratings to neutral and stressful images throughout exposure (left) and over time (right). Baseline = response to gray screen. (b) Stress regulation skills as assessed by DERS questionnaire. Higher values = greater difficulties. **p < .01; ***p < .001. DERS = Difficulties in Emotion Regulation Scale. Error bars = ± 1 SE.

Emotional stress reactivity was determined from participant ratings of subjective stress throughout the fMRI scan (Fig. 1a). There was a significant main effect of condition (stress vs neutral, controlling for condition order) on self-reports of stress (*F*(1,58) = 166.51, *p* < .001), demonstrating that the stressor successfully evoked an emotional response. Pre-existing anxiety levels did not correlate with stress reactivity (all: *r*(59) = 0.17, *p* = .19; male: *r*(29) = −0.1, *p* > .25, female: *r*(30) = 0.3, *p* = .1). There were no significant main effects of sex (*F*(1,57) = 0.78, *p* > .25). There was a trend-level sex × condition interaction (*F*(1,58) = 3.25, *p* = .08), although both male and female participants showed a robust effect of condition (male: *F*(1,28) = 74.14, *p* < .001; female: *F*(1,30) = 93.01, *p* < .001), demonstrating that both groups had emotional responses to the stressor. However, although the effect of stressor exposure did not significantly change over time (condition x time: *F*(2,116) = 1.6, *p* = .21; condition x time x sex: *F*(2,116) = 1.24, *p* > .25), there was a significant difference between male and female participants’ responses over time, with females showing larger changes (sex x time: *F*(2,116) = 4.1, *p* = .019). Together with prior work showing an evolving neural response to stress over time using a similar paradigm (Sinha et al., 2016), this time effect led us to include time in our analysis of brain responses to stress.

Stress regulation abilities were assessed using the DERS (Fig. 1b). Consistent with previous reports (recently reviewed in Young et al., 2019), pre-existing anxiety levels correlated with stress regulation abilities (total DERS score, all: *r*(56) = 0.63, *p* < .001; male: *r*(27) = 0.699, *p* < .001; female: *r*(27) = 0.65, *p* < .001). However, there were no significant differences between male and female participants in total DERS score (independent samples *t*-test; *t*(57) = -0.33, *p* > .25) or five of the six DERS subscales (nonacceptance: *t*(57) =- 0.55, *p* > .25; goals: *t*(57) = 0.26, *p* > .25; impulse: *t*(57) = 1.33, *p* = .19; strategies: *t*(57) = 1.07, *p* > .25; clarity: *t*(57) = -0.25, *p* > .25). Male and female participants did differ significantly in emotional awareness, with males reporting significantly lower emotional awareness than females (*t*(57) = -3.41, *p* = .001). Demonstrating the independence of these emotional assays, there were no significant correlations between DERS scores (total or any subscales) and emotional reactivity to stress in the moment (subjective stress ratings during stress relative to baseline, all *p* > .18).

### 3.2 Neural response to stressor exposure

**Fig. 2:**
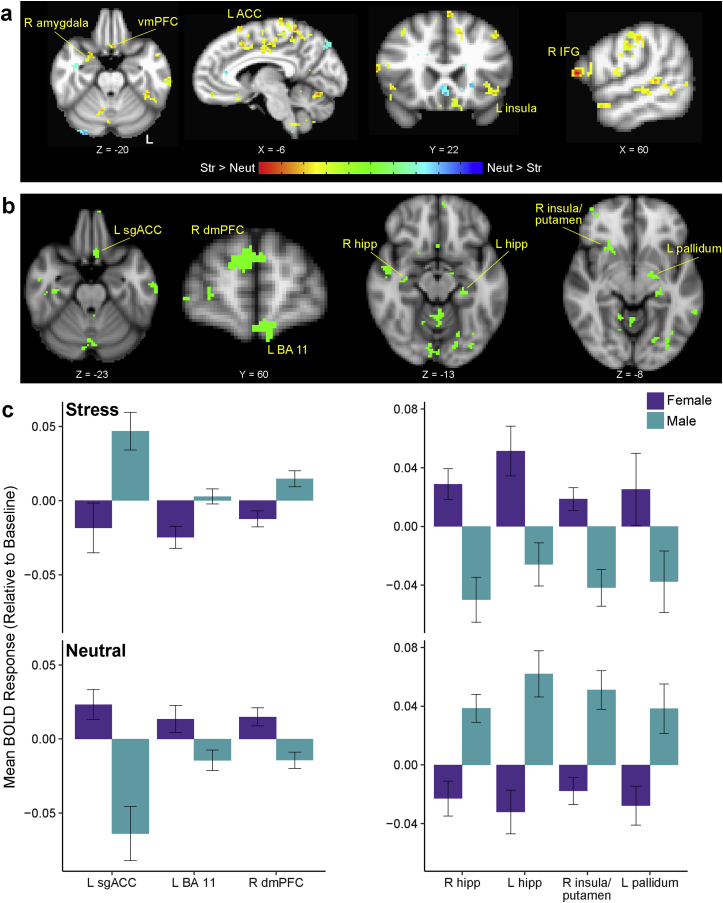
Responses to sustained stressor exposure: general and sex-specific. (a) Regions showing overall main effect of Condition (Stress vs Neutral). (b) Regions showing significant Condition (Stress vs Neutral) by Sex (Female vs Male) interaction. (c) Mean BOLD responses for male and female participants within clusters shown in (b) in each condition. Error bars = ± 1 SE. Conditions: Neut = Neutral, Str = Stress. Regions: ACC = anterior cingulate cortex; IFG = inferior frontal gyrus; vmPFC = ventromedial prefrontal cortex; sgACC = subgenual anterior cingulate cortex; dmPFC = dorsomedial prefrontal cortex. All brain images thresholded at voxelwise *p* < .01, cluster-corrected α = 0.05.

Using a linear mixed effects model (AFNI's 3dLME) with condition, time, and sex as predictors and subject as a random effect, we ran a whole brain analysis to investigate which regions showed significant blood oxygen level dependent (BOLD) responses to stressful relative to neutral image exposure. Consistent with previous findings, there were significant increases in signal during the stress relative to neutral condition in regions including anterior cingulate cortex, orbitofrontal cortex, vmPFC, inferior frontal gyrus, midtemporal regions, insula, amygdala, and lateral occipital cortex (Fig. 2a; main effect of condition; *p* < .01, cluster-corrected α = 0.05; all significant gray matter clusters shown in Table S1). At this statistical threshold, no regions showed significant condition × time or sex × time interactions.

In addition to these overall effects of stress, a condition × sex interaction revealed several regions showing sex-specific stress responses (full list in Table S2). Overall, male participants had heightened prefrontal (particularly in medial prefrontal regions) and blunted limbic/striatal responses to prolonged stress exposure. In contrast, female participants had blunted prefrontal and higher limbic/striatal responses (Fig. 2b–c).

### 3.3 Sex differences in relationships between neural stress responses and stress reactivity and regulation

We extracted the mean BOLD response for each participant during stress relative to baseline for clusters that showed sex-specific stress responses (i.e., significant condition × sex interaction). Based on prior literature (see Introduction), we examined medial prefrontal (dmPFC and ventromedial regions sgACC and BA 11) and limbic/striatal regions (hippocampus, putamen, pallidum). We ran linear models using BOLD response and sex as predictors to test whether BOLD responses in these sex-specific stress regions were associated with differences in stress reactivity during the fMRI scan and stress regulation as measured by DERS.

#### 3.3.1 Stress reactivity

**Fig. 3:**
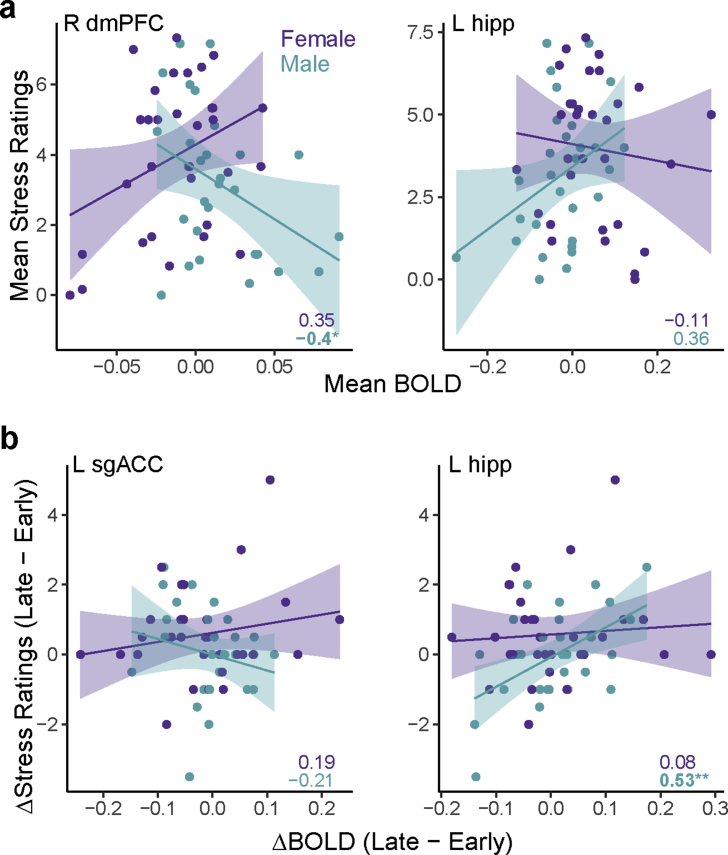
Sex-specific neural stress responses are differentially associated with emotional stress reactivity for males and females. Numbers indicate Pearson's r values for male and female participants separately. (a) Responses in dmPFC and hippocampus throughout sustained stress exposure differentially predict stress reactivity: higher dmPFC responses were associated with greater subjective stress for females and lower stress for males, whereas higher hippocampal responses had the opposite pattern. Mean BOLD responses were computed as the average response to the Stress condition (6 blocks) – Baseline (2 Gray blocks). (b) Changes in ventromedial PFC response (subgenual anterior cingulate, sgACC) throughout stress exposure were associated with changes in subjective stress, with increasing responses associated with decreasing subjective stress for males but increasing subjective stress for females. Changes in left hippocampal responses showed the opposite pattern. Change in BOLD was computed as the difference between Late (last 2 Stress blocks) – Early (first 2 Stress blocks), each relative to Baseline. dmPFC = dorsomedial prefrontal cortex; hipp = hippocampus. Regions shown in Fig. 2b. *p < .05.

We first investigated whether sex-specific stress responses in the corticolimbic/striatal network would differentially predict emotional reactivity during the stressor (Fig. 3). We found a significant interaction between BOLD responses in right dmPFC and sex (β: M = −53.86 [SE = 17.84], *p*  =  .004). For males, who had overall higher responses in dmPFC during stress compared to females, these higher responses corresponded with lower stress reactivity, whereas for females, higher dmPFC responses were associated with greater reactivity. This pattern was not observed in ventromedial PFC regions (BOLD x Sex: *p* > .2). However, we found the opposite pattern in the left hippocampus (BOLD x Sex x ROI [R dmPFC vs L hipp]: β = -65.94 [19.26], *p* < .001. Although females had overall higher hippocampal responses to stress in these regions, higher responses corresponded to higher stress reactivity for males but not females (BOLD x Sex: β = 12.08 [6.39], *p* = .064). This pattern was not observed in the more anterior right hippocampal region (BOLD x Sex: *p* > .2). Main effects of BOLD and Sex were not statistically significant in any of these models.

We ran an exploratory analysis to test whether dynamic changes in neural responses to stress were associated with changes in emotional stress reactivity. Specifically, we tested whether there were any relationships between changes in subjective stress ratings (Late [average of last 2 runs] – Early [first 2 runs], relative to baseline [2 Gray runs]) scores and the magnitude of change in any of our ROIs throughout stress exposure (Late – Early, relative to baseline). Consistent with average responses throughout the stressor reported above (Fig. 3a), dynamic increases in left hippocampus responses were associated with increasing subjective stress for males and decreasing subjective stress for females (ΔBOLD x Sex: β = 7.45 [3.69], *p* = .048). However, we saw the opposite pattern in sgACC (ΔBOLD x Sex x ROI [L hipp vs L sgACC]: β = -14.56 [6.05], *p* = .018). For males, increasing sgACC responses over time were associated with decreases in subjective stress ratings, but not for females( ΔBOLD x Sex: β = −7.12 [4.83], *p* = .15). These patterns were not observed in dmPFC or right hippocampus (*p* > .25). Again, main effects of BOLD and Sex were not statistically significant. All interactions remained significant when controlling for anxiety levels.

#### 3.3.2 Stress regulation

**Fig. 4:**
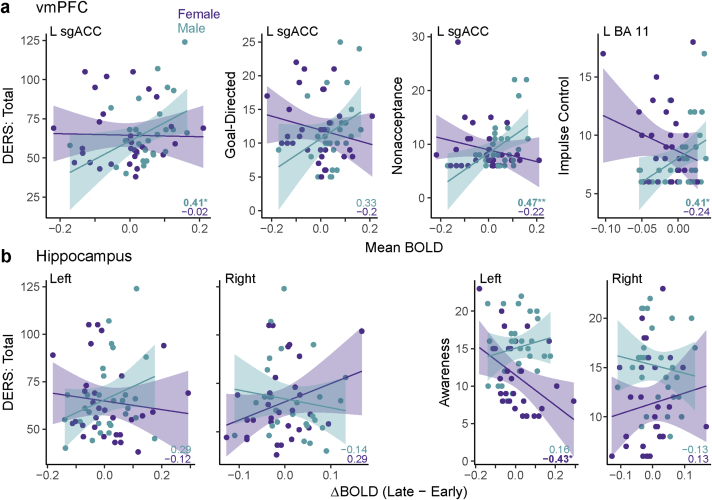
Sex-specific neural stress responses are differentially associated with stress regulation for males and females. Numbers indicate Pearson's r values for male and female participants separately. (a) Responses in vmPFC (ventromedial prefrontal cortex) regions throughout stress differentially predict DERS scores, with higher responses correlating with worse emotion regulation for males and better emotion regulation for females. This pattern was significant for the Difficulties Engaging in Goal-Directed Behavior, Nonacceptance of Emotional Responses, and Impulse Control Difficulties subscales. As in Fig. 3, BOLD responses were computed as the average response to the Stress condition (6 blocks) – Baseline. (b) Changes in hippocampal BOLD throughout stress were differentially associated with DERS for left and right regions. Increasing responses in left hippocampus correlated with worse emotion regulation for males and better emotion regulation for females, whereas increasing responses in right hippocampus correlated with better regulation for males and worse regulation for females. This pattern was significant for the Lack of Emotional Awareness subscales. As in Fig. 3, change in BOLD responses was computed as Late (last 2 blocks) – Early (first 2 blocks) of stress exposure, each relative to Baseline . *p < .05, **p < .01.

Having shown that sex-specific stress responses in the corticolimbic/striatal circuit differentially related to stress reactivity, we examined whether these responses were also differentially associated with stress regulation (Fig. 4). We found a trend-level left sgACC BOLD × sex interaction predicting total DERS scores (β = 121.97 [65.23], *p* = .067), but no significant main effects of BOLD or sex (*p* > .2). Although male participants on average showed higher sgACC responses to stressor exposure than females, higher signal in this region was associated with worse emotion regulation for males, but better emotion regulation for females. This pattern did not exist in dorsomedial PFC (*p* > .25). We then ran an exploratory analysis to test whether dynamic changes in neural responses to stress were associated with stress regulation abilities. Specifically, we tested whether there were any relationships between DERS scores and the magnitude of change in any of our ROIs throughout stress exposure (again, late – early, each relative to baseline). Although the changes in prefrontal regions were not associated with DERS, we found that the change in hippocampal response over time predicted total DERS. Notably, this association differed between left and right hippocampus (ΔBOLD x Sex x ROI [L hipp vs R hipp]: β =-232.39 [104.92], *p* = .029, still significant when controlling for anxiety). In left hippocampus, males who had increasing responses throughout stressor exposure also had worse emotion regulation (ΔBOLD x Sex: β = 92.21 [57.92], p = .12), but in right hippocampus, females who had increased responses had worse emotion regulation (ΔBOLD x Sex: β = -140.19 [87.44], *p* = .12).

We then investigated which components of stress regulation were associated with the significant interactions between sex and stress-induced brain responses. There was a significant sgACC BOLD × sex interaction for the DERS subscales of difficulties engaging in goal-directed behavior (β = 34.6 [16.42], *p* = .04) and nonacceptance of emotional responses (β = 41.95 [14.79], *p* = .006). There was also a significant BOLD x sex interaction predicting impulse control difficulties in another vmPFC region, BA 11 (β = 70.14 [28.43], *p* = .017). In contrast, the hippocampal laterality x ΔBOLD x sex interaction significantly predicted lack of emotional awareness (β = -47.11 [23], *p* = .043; left hipp ΔBOLD x Sex: β = 28.17 [12.32], *p* = .026; right hipp: *p* > .2 ). There was also a significant main effect of sex on emotional awareness (β = 3.58 [1.09], *p* = .002); as mentioned earlier, male participants reported significantly lower emotional awareness than female participants. Finally, similar to the pattern in sgACC, there was a significant ΔBOLD x sex interaction in the left hippocampus predicting nonacceptance of emotional responses (β = 30.05 [13.22], *p* = .027).

## 4 Discussion

Both stress reactivity and regulation are core components of adaptive stress responses and have been shown to differ between men and women. In this experiment, we extend prior work by revealing brain regions that show significantly different responses during stress for men and women. By relating these unique patterns of activity to emotional stress responses, we further demonstrate that men and women engage distinct networks to facilitate optimal stress reactivity and regulation.

Across all participants, stressor exposure led to increased levels of emotional stress reactivity as well as changes in BOLD responses throughout the brain. Consistent with previous reports, we found stress-related increases in regions including the salience network (amygdala, temporal pole, dorsal anterior cingulate) as well as medial PFC and posterior cingulate (Seo et al., 2011; Sinha et al., 2004; Sinha et al., 2016; van Oort et al., 2017). We also found higher responses in primary visual regions which, together with increased responses in the salience network, have been posited to reflect hypervigilance and potentiated visual processing (Henckens et al., 2009). As with other (non-personalized) stressors, we found overall decreases in hippocampal signal (Dedovic, D'Aguiar and Pruessner, 2009).

In addition to these overall effects of stressor exposure, we found several regions throughout the brain for which male and female participants showed distinct responses during stressor exposure. This analysis of interactions between sex and stressor exposure provides an important validation and extension of previous findings of sex-specific stress responses. First, we used a whole-brain analysis with a strict significance threshold, bolstering findings from ROI-based analyses (e.g., Goldstein et al., 2010; Kogler et al., 2015a). Second, this analysis revealed sex-specific responses during stressor exposure that were not modulated by individual differences in emotional stress reactivity (Seo et al., 2017; Wang et al., 2007). Indeed, as we did not find significant differences between men and women in overall subjective stress ratings, these neural responses were not simply the result of differences in emotional stress reactivity. Although women are often shown to self-report higher levels of subjective stress than men, the use of a non-psychosocial stressor in the current experiment may have mitigated these differences (Stroud et al., 2002).

Throughout stressor exposure, men showed higher responses in prefrontal regions, whereas women had higher responses in limbic/striatal areas. Such widespread differences are consistent with the influence of sex hormones (including estrogen and androgen) throughout the brain, as well as evidence from nonhuman animals of sex-specific stress effects on prefrontal and limbic structure and function (McEwen and Milner, 2017). In particular, higher PFC responses in men are consistent with prior stress findings from human neuroimaging studies (Seo et al., 2011; Wang et al., 2007), and work showing that men engage these regions more when cognitively regulating emotional responses (Domes et al., 2010). We found distinct associations between emotional stress responses and medial prefrontal stress responses along the dorsal/ventral axis, extending findings that these subregions differentially mediate hypothalamic stress responses (Radley et al., 2006). For dorsomedial PFC (dmPFC), we found that higher stress-induced responses were associated with greater stress reactivity for women, but lower stress reactivity for men. This is consistent with work showing that dmPFC responses during stress positively correlated with subjective anxiety for women, but negatively correlated with subjective anxiety for men (Seo et al., 2017). This may reflect differences in emotion regulation strategies, as high responses in this region to unavoidable threats have been associated with greater avoidance of immediate loss (Schlund et al., 2015).

In contrast to high dmPFC stress responses supporting lower stress reactivity, high stress responses in ventromedial PFC (vmPFC) regions were maladaptive for men. Higher vmPFC responses were associated with worse goal-directed behavior, acceptance of emotional responses, and impulse control for men, but improvements in these dimensions of stress regulation for women. The association between vmPFC and impulsivity/goal-directed control is consistent with prior work demonstrating financial impulsivity, or heightened sensitivity to immediate rewards (McClure et al., 2004). A recent large-scale study reported that high vmPFC stress responses were associated with high subjective stress responses, although this study did not examine whether this relationship differed between male and female participants (Orem et al., 2019). However, unlike overall high stress-induced responses, dynamic increases in vmPFC responses during stress (particularly subgenual anterior cingulate, sgACC) were beneficial for men, and were associated with decreases in emotional reactivity during stress. These results suggest that plasticity in this region, particularly for men, helped support in-the-moment regulation of these emotional responses. This is consistent with evidence that the vmPFC dynamically tracks subjective appraisals of stimulus value (McGuire and Kable, 2015) as well as the positive association between increasing vmPFC responses during stress and use of adaptive coping strategies (Sinha et al., 2016).

Although we did not find higher responses in the amygdala for women as hypothesized, we did observe that women had significantly higher bilateral hippocampal responses to stress than men. Higher hippocampal responses in women have also been shown in studies examining negative emotion (Stevens and Hamann, 2012), and may be related to reports of stronger long-term memory for emotional experiences in women (Ferree and Cahill, 2009; Hsu et al., 2018). This result is also noteworthy given evidence that the effects of stress on memory differ as a function of sex (Shields et al., 2017). Hippocampal responses also appeared to play more adaptive roles in emotional stress responses for women. For men, overall high and dynamically increasing stress responses in the left hippocampus were associated with higher stress reactivity, and dynamic increases were also associated with worse stress regulation. The maladaptive effects of *increasing* hippocampal responses during stress in men mirror prior findings that *decreasing* hippocampal responses were associated with an adaptive stress response (Sinha et al., 2016). Notably, sex differences in the relationship between hippocampal signal and stress regulation (both overall and in awareness of emotions) varied between right and left hippocampus. The finding that women showed improved stress reactivity and regulation with responses from left (but not right) hippocampus may be related to left hippocampal involvement in verbal memory (Kelley et al., 1998) and greater use of verbal emotion regulation strategies by women (Tamres et al., 2002). Such laterality-specific relationships are also consistent with prior findings: in men, higher gray matter volume in a region including left hippocampus was associated with worse emotion regulation (Kong et al., 2014), and in women, higher right hippocampal responses to emotion regulation were associated with higher subjective stress (Kogler et al., 2015a).

We also observed significantly higher stress-induced responses for women in anterior insula and putamen. Prior studies of sex differences in stress and emotion reactivity in these regions have been mixed. For example, studies of emotion perception and experience have similarly reported that women had higher responses in these regions (Whittle et al., 2011). However, there are also reports of higher putamen responses in men than women during (Kogler et al., 2015a) and after stress (Lighthall et al., 2012). Several methodological differences, including the type of stressor (Kogler et al., 2015b) and the delay between stressor onset and assessment of brain responses, may underlie these divergent results. Understanding dorsal striatal responses to stress exposure is particularly important as stress is thought to promote maladaptive behaviors through increased reliance on dorsolateral striatum-dependent habits (Packard, 2009). However, although acute stress-induced shifts toward striatal memory have been demonstrated in male rodents (Kim et al., 2001; Packard and Wingard, 2004; Siller-Perez et al., 2017), men (Vogel et al., 2017), and mixed-sex human cohorts (Goldfarb et al., 2017; Schwabe and Wolf, 2012), there have been reports of the opposite pattern in women (Schwabe et al., 2009). Thus, more work is needed to characterize the effects of stress on striatal processes in women. Our observation that women showed heightened insular activity under stress, which has been associated with emotional awareness (Craig, 2009), may indicate greater attention by women toward their emotional state under stress (Pais-Vieira et al., 2016) and is consistent with the higher emotional awareness reported by the women in our sample. However, we did not observe a significant relationship between the magnitude of insular response and levels of emotional awareness among females.

Together, these results reveal that men and women engage distinct corticolimbic/striatal networks during brief sustained stressor exposure. A limitation of this study is that we did not have sufficient power to separate women by phase of menstrual cycle. Menstrual phase has been shown to influence patterns of stress-induced brain responses (Goldstein et al., 2010), and levels of sex hormones have been associated with differences in stress-induced physiological responses (Juster et al., 2016). Although we were not able to compare the effects of different hormonal contraceptives (as in Herrera et al., 2019), a preliminary comparison between women taking no contraceptives and those taking oral contraceptives indicated that neural and emotional responses, as well as the reported brain/emotion associations, were largely consistent (Fig. S1). Whether the relationship between stress-induced brain responses and emotional stress responses differs by menstrual cycle phase and sex hormone levels (in both sexes) remains a question for future research. For example, low levels of free androgens have been associated with greater stress-induced vmPFC responses in a clinical population of males (Goldstein et al., 2015), although how these relate to emotional stress responses is unclear. We further demonstrate that, not only do men and women engage different networks during stress, these corticolimbic/striatal regions also differentially relate to emotional stress responses for men and women. As men and women did not differ significantly in emotional stress responses, these sex-specific brain/emotion associations provide an example of “sex convergence” (distinct mechanisms toward a similar endpoint; Bangasser and Wicks, 2017). For men, increasing (but not overall high) responses to stress in vmPFC correspond to adaptive emotional responses, whereas for women, both increasing and overall high left hippocampal stress responses correspond to adaptive emotional responding. These distinct patterns reveal important sex differences underlying optimal emotional stress reactivity and regulation.
