# Supplementary material for: Sex differences in neural stress responses and correlation with subjective stress and stress regulation
Source: Neurobiol Stress. 2019 May 25;11:100177. doi: 10.1016/j.ynstr.2019.100177 (PMC6603439; doi:10.1016/j.ynstr.2019.100177)
Supplement: Multimedia component 1 [file mmc1.docx]

**Sex Differences in Neural Stress Responses and**

**Correlation with Subjective Stress and Stress Regulation**

Elizabeth V. Goldfarb, Dongju Seo & Rajita Sinha

**Supplementary Tables**

**Table S1. Regions showing significant main effect of Condition (Stress vs. Neutral)**

|  | **Lat** | **BA** | **X** | **Y** | **Z** | **Size**  **(Voxels)** | **F value**  **M [SEM]** |
| --- | --- | --- | --- | --- | --- | --- | --- |
| **STRESS > NEUTRAL** |  |  |  |  |  |  |  |
| ***Frontal*** |  |  |  |  |  |  |  |
| Frontal pole | L | 9 | -22 | 46 | 36 | 155 | 10.52 [0.23] |
|  | R | 10 | 29 | 54 | 1 | 78 | 13.18 [0.59] |
|  | R | 8 | 21 | 40 | 46 | 90 | 12.58 [0.6] |
| Frontal pole (dlPFC) | R | 9 | 25 | 51 | 38 | 101 | 11.18 [0.39] |
| vmPFC | Bilat | 11 | 2 | 49 | -15 | 106 | 11.91 [0.46] |
| sgACC/OFC | L | 11/25 | -6 | 20 | -21 | 56 | 11.33 [0.72] |
| OFC/frontal operculum/insula | L | 47 | -40 | 22 | -6 | 58 | 11.51 [0.55] |
| OFC/inferior frontal gyrus | R | 45 | 38 | 29 | 0 | 101 | 11.08 [0.37] |
| Paracingulate cortex | R | 10 | 10 | 54 | 11 | 167 | 11.64 [0.35] |
| Superior frontal gyrus | L | 6 | -4 | 21 | 63 | 63 | 12.29 [0.51] |
|  | R | 9 | 7 | 46 | 33 | 72 | 12 [0.56] |
|  |  | 8 | 7 | 35 | 48 | 96 | 11.24 [0.5] |
| Middle frontal gyrus | L | 8 | -41 | 26 | 44 | 62 | 11.35 [0.51] |
|  | R | 8 | 35 | 25 | 50 | 59 | 11.6 [0.6] |
| Frontal pole/inferior frontal gyrus | R | 45 | 54 | 35 | 1 | 64 | 12.26 [0.57] |
| Inferior frontal gyrus | L | 44 | -51 | 10 | 23 | 104 | 10.23 [0.32] |
|  | R | 44 | 49 | 13 | 26 | 124 | 12.54 [0.48] |
|  |  | 44/9 | 57 | 20 | 15 | 138 | 13.27 [0.62] |
| ACC/precentral/postcentral | Bilat | 5/6 | -2 | -18 | 58 | 1721 | 12.05 [0.12] |
| Precentral gyrus | L | 6 | -20 | -10 | 66 | 140 | 12.27 [0.41] |
|  | R | 4 | 10 | -29 | 72 | 62 | 11.5 [0.64] |
| ***Temporal*** |  |  |  |  |  |  |  |
| Temporal pole | L | 22 | -56 | 4 | -12 | 149 | 12.31 [0.43] |
|  | R | 38 | 56 | 11 | -15 | 65 | 12.67 [0.66] |
| Planum temporale/central opercular cortex/middle temporal gyrus | L | 41 | -57 | -17 | 6 | 506 | 11.6 [0.2] |
| Planum temporale/ parietal opercular cortex | R | 40/41 | 48 | -30 | 19 | 177 | 13.16 [0.46] |
| Middle temporal gyrus | L | 21 | -60 | -22 | -21 | 60 | 12.55 [0.73] |
| Middle temporal gyrus |  | 21 | -62 | -36 | -2 | 185 | 12.22 [0.42] |
| Middle/superior temporal gyrus | R | 21/22 | 56 | -33 | 2 | 203 | 11.87 [0.31] |
| Temporal fusiform cortex | L | 37/19 | -36 | -41 | -21 | 260 | 11.71 [0.31] |
| ***Parietal*** |  |  |  |  |  |  |  |
| Central operculum/postcentral gyrus | R | 1 | 55 | -7 | 16 | 79 | 11.37 [0.48] |
| Postcentral gyrus | R | 4 | 52 | -15 | 38 | 287 | 11.39 [0.27] |
| Postcentral/supramarginal gyrus | L | 40/1 | -53 | -19 | 35 | 336 | 11.26 [0.23] |
|  | R | 1 | 50 | -27 | 41 | 80 | 13.9 [0.7] |
| Precentral/postcentral/  superior parietal lobule | L |  | -33 | -35 | 59 | 249 | 11.31 [0.31] |
|  | R |  | 33 | -25 | 58 | 868 | 12.39 [0.17] |
| Precentral/postcentral/supramarginal gyrus | L | 1/4 | -35 | -27 | 41 | 142 | 12.11 [0.43] |
| Parietal operculum/insula | L | 1/13 | -38 | -29 | 19 | 162 | 12.27 [0.4] |
| Superior parietal lobule | L | 7 | -16 | -56 | 61 | 66 | 10.62 [0.38] |
| Precuneus | Bilat | 31 | 3 | -63 | 17 | 75 | 11.87 [0.65] |
| LO | R | 39/7 | 34 | -69 | 45 | 158 | 10.61 [0.3] |
| ***Occipital*** |  |  |  |  |  |  |  |
| LO | L | 19 | -50 | -64 | 3 | 342 | 12.89 [0.33] |
|  |  | 19 | -42 | -75 | 13 | 125 | 11.81 [0.47] |
|  |  | 19 | -24 | -79 | 30 | 93 | 11.54 [0.42] |
|  | R | 19/37 | 45 | -65 | 2 | 700 | 12.86 [0.23] |
| WM/occipital fusiform | R |  | 29 | -77 | 4 | 76 | 13.52 [0.68] |
| LO/cuneal cortex | R | 19 | 20 | -80 | 31 | 87 | 11.22 [0.44] |
| Occipital fusiform/LO | L | 19 | -34 | -83 | -14 | 77 | 12.25 [0.56] |
| ***Subcortical*** |  |  |  |  |  |  |  |
| Thalamus/hippocampus | L | 50 | -19 | -31 | 1 | 141 | 11.61 [0.4] |
| Amygdala | R | 53 | 24 | 5 | -24 | 66 | 11.69 [0.56] |
| ***Cerebellum*** |  |  |  |  |  |  |  |
| Lingual gyrus/cerebellum | R |  | 10 | -63 | -12 | 531 | 11.82 [0.2] |
| VIIIa/IX | L |  | -10 | -60 | -51 | 229 | 12.03 [0.32] |
| VIIIa | L |  | -33 | -44 | -47 | 108 | 14.05 [0.78] |
| VIIIa | R |  | 9 | -70 | -42 | 112 | 13.56 [0.73] |
| **NEUTRAL > STRESS** |  |  |  |  |  |  |  |
| **Frontal** |  |  |  |  |  |  |  |
| Anterior cingulate | L | 24 | -5 | 34 | 12 | 60 | 11.36 [0.44] |
| Subcallosal cortex | R | 32 | 0 | 28 | -5 | 87 | 12.52 [0.6] |
| ***Parietal*** |  |  |  |  |  |  |  |
| Supramarginal gyrus | L | 40 | -54 | -33 | 51 | 66 | 14.37 [0.76] |
| Precuneus/LO | L | 7 | -9 | -79 | 51 | 105 | 10.02 [0.26] |
| ***Occipital*** |  |  |  |  |  |  |  |
| Cuneal cortex | R | 18 | 4 | -79 | 36 | 63 | 11.68 [0.54] |
| ***Subcortical*** |  |  |  |  |  |  |  |
| Hippocampus/WM | R |  | 39 | -15 | -17 | 200 | 12.12 [0.39] |
| Putamen | L | 49 | -22 | 18 | -3 | 70 | 14.38 [0.77] |
| Pallidum/putamen | R |  | 16 | -1 | -1 | 101 | 11.56 [0.42] |
| Caudate | L | 48 | -13 | 13 | 15 | 58 | 11.34 [0.51] |
| ***Brainstem*** |  |  |  |  |  |  |  |
| Brainstem | R |  | 1 | -45 | -67 | 58 | 12.01 [0.64] |
| ***Cerebellum*** |  |  |  |  |  |  |  |
| Brainstem/cerebellum | L |  | -13 | -37 | -42 | 63 | 12.18 [0.55] |
| Crus I | R |  | 31 | -84 | -24 | 74 | 12.85 [0.6] |
| Crus II | R |  | 44 | -66 | -46 | 62 | 10.4 [0.39] |

MNI coordinates shown for center of mass of significant clusters. Voxelwise *p* < .01, cluster-corrected α = .05.

**Table S2. Regions showing significant Condition (Stress vs. Neutral) x Sex (Female vs. Male) Interaction**

|  | **Lat** | **BA** | **X** | **Y** | **Z** | **Size (Voxels)** | **F value**  **M [SEM]** |
| --- | --- | --- | --- | --- | --- | --- | --- |
| ***Frontal*** |  |  |  |  |  |  |  |
| mPFC (BA 11) | L | 11 | -5 | 59 | -18 | 64 | 11.37 [0.5] |
| dmPFC | R | 9/10 | 8 | 57 | 24 | 306 | 11.93 [0.26] |
| Frontal pole | L | 10 | -28 | 40 | 26 | 115 | 13.23 [0.52] |
|  | R | 10 | 35 | 54 | -1 | 122 | 11.04 [0.38] |
|  |  | 10 | 38 | 48 | 23 | 60 | 10.52 [0.4] |
|  |  | 9 | 37 | 37 | 38 | 63 | 11.28 [0.48] |
| sgACC | L | 25 | -2 | 18 | -21 | 70 | 13.76 [0.99] |
| Middle frontal gyrus | L | 6 | -30 | 1 | 50 | 131 | 12.38 [0.44] |
|  | R | 8 | 35 | 25 | 46 | 66 | 12.27 [0.75] |
| WM/inferior frontal gyrus | L | 45 | -31 | 22 | 16 | 56 | 9.85 [0.34] |
| Inferior frontal gyrus | R | 44 | 43 | 17 | 24 | 74 | 12.2 [0.57] |
| Supplementary motor cortex | R | 24 | 14 | -12 | 50 | 71 | 11.35 [0.57] |
| Precentral gyrus | L | 6 | -27 | -15 | 71 | 107 | 11.54 [0.42] |
|  | R | 6 | 9 | -25 | 56 | 68 | 12.96 [0.72] |
| Pre/postcentral gyrus | L | 4 | -34 | -20 | 57 | 72 | 13.94 [0.85] |
| Postcentral gyrus | L | 1/4 | -61 | -13 | 34 | 61 | 13.07 [0.73] |
|  | L | 1 | -48 | -20 | 48 | 57 | 11.82 [0.55] |
| ***Temporal*** |  |  |  |  |  |  |  |
| Middle temporal gyrus | L | 21 | -64 | -42 | 2 | 159 | 13.63 [0.53] |
|  | R | 21 | 65 | -46 | 8 | 61 | 11.93 [0.59] |
| Middle temporal gyrus, posterior | L | 21 | -62 | -16 | -22 | 84 | 12.08 [0.45] |
| Middle/superior temporal gyus | R | 21/22 | 52 | -8 | -17 | 64 | 13.72 [0.81] |
| WM/superior temporal gyrus | R |  | 40 | -32 | 5 | 64 | 11.82 [0.58] |
| Planum temporale | R | 40 | 62 | -20 | 12 | 142 | 12.13 [0.42] |
| ***Parietal*** |  |  |  |  |  |  |  |
| Parietal operculum cortex | L | 40/1 | -33 | -29 | 18 | 56 | 11.42 [0.48] |
| Supramarginal gyrus | L | 40 | -39 | -38 | 37 | 63 | 12.69 [0.77] |
|  |  | 39 | -64 | -46 | 32 | 61 | 11.71 [0.54] |
| Posterior cingulate cortex/precuneus | L | 23/31 | -10 | -52 | 28 | 113 | 11.95 [0.46] |
| Angular gyrus/LO | L | 39 | -44 | -57 | 54 | 88 | 11.45 [0.36] |
| Lateral occipital cortex | L | 39 | -49 | -63 | 18 | 84 | 11.73 [0.47] |
|  | L | 39 | -35 | -71 | 43 | 133 | 11.87 [0.44] |
|  | R | 19/39 | 47 | -62 | 19 | 67 | 10.84 [0.46] |
|  | R | 19 | 43 | -77 | 23 | 65 | 10.72 [0.58] |
|  | R | 19 | 28 | -82 | 32 | 102 | 12.46 [0.58] |
|  | R | 7 | 21 | -68 | 53 | 75 | 11.27 [0.45] |
| ***Occipital*** |  |  |  |  |  |  |  |
| Precuneus/cuneal cortex | L | 18 | -5 | -73 | 31 | 97 | 10.22 [0.33] |
| Intracalcarine cortex | L | 17 | -23 | -73 | 7 | 56 | 10.55 [0.49] |
| LO/occipital fusiform | L | 18/19 | -34 | -75 | -7 | 76 | 11.37 [0.45] |
| Occipital fusiform gyrus | L | 18 | -19 | -78 | -13 | 82 | 11.1 [0.41] |
| LO | L | 19 | -36 | -75 | 27 | 72 | 11.59 [0.59] |
| LO/occipital pole | R | 18/19 | 13 | -87 | 23 | 90 | 11.91 [0.53] |
| Lingual gyrus | R |  | 6 | -85 | -17 | 90 | 13.31 [0.64] |
| ***Subcortical*** |  |  |  |  |  |  |  |
| Insula/putamen | R | 49 | 25 | 18 | -6 | 86 | 12.15 [0.55] |
| Pallidum | L |  | -17 | -9 | -10 | 57 | 11.87 [0.78] |
| Hippocampus | L |  | -26 | -26 | -10 | 83 | 10.73 [0.47] |
|  | R | 54 | 39 | -20 | -19 | 59 | 11.89 [0.67] |
| ***Cerebellum*** |  |  |  |  |  |  |  |
| I-IV/Lingual gyrus | Bilat | 0 | 4 | -54 | -11 | 189 | 12.66 [0.4] |
| Crus II/VIIb | L | 0 | -16 | -73 | -43 | 57 | 11.24 [0.51] |
| Vermis VI | R | 0 | 3 | -74 | -24 | 56 | 10.54 [0.44] |

MNI coordinates shown for center of mass of significant clusters. Voxelwise *p* < .01, cluster-corrected α = .05.


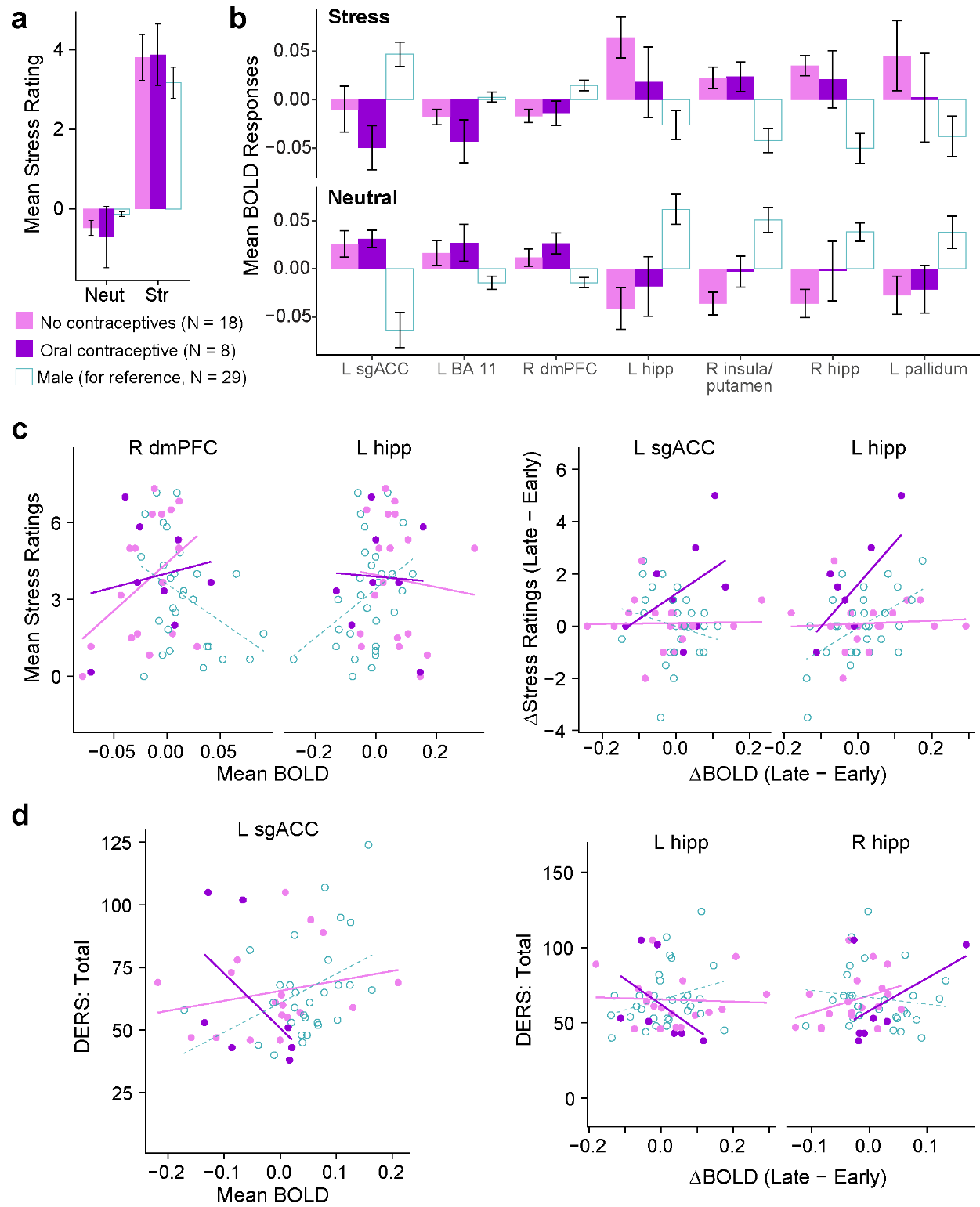

Fig. S1. Preliminary comparison of neural stress responses and stress reactivity/regulation by contraceptive status. (a) Stress reactivity (and regulation via DERS scores, not shown) did not differ between women taking no contraceptives and those on oral contraceptives (OC, all *p* > .25, see Main Text Fig. 1). (b) Neural stress responses do not significantly differ based on contraceptive status (frontal regions: all *p* > .19; subcortical: all *p* > .25, see Fig. 2). (c) Relationships between neural stress responses and stress reactivity. The relationship between overall BOLD response and stress ratings did not differ by contraceptive status (BOLD x contraceptive interaction: all *p* > .25), but the exploratory association between change in hippocampal BOLD and change in ratings was more positive for women taking oral contraceptives (β = 15.62 [6.38], *p* = .023; Fig. 3). (d) Relationships between neural stress responses and stress regulation did not significantly differ by contraceptive status (Fig. 4; change in BOLD x contraceptive: *p* > .19), although there was a trend-level difference in the relationship between sgACC BOLD and total DERS (β = -258.46 [127.1], *p* = .055).
